# 

**Published:** 1883-05

**Authors:** 


### Pamphlets.

---

Is gonorrhœa a bacteria disease? Some investigations carried on in the out-door department of surgery of the

University Hospital, Baltimore. By Newberry A. S. Keyser. Reprint from the Maryland *Medical Journal.*

The treatment of acute oczema. By George H. Rohe, M.D., Baltimore.

The electric light in surgical diagnosis. By Roswell Park, M.D., Chicago. Reprint from the *Annals of Anatomy and Surgery.*

Secondary batteries and the so-called storage of electricity. By Roswell Park, M. D. Reprint from the Chicago *Medical Journal and Examiner.*

Modified Listerism in ovariotomy, with a report of five recent operations. By Edward W. Jenks, Chicago. Reprint from the Michigan *Medical News.*

Bacteria and their presence in syphilitic secretions. From the clinic of Professor Newman, Vienna. By Robert B. Morison, M.D., Baltimore. Reprint from the Maryland *Medical Journal.*

The charge of exclusioism, as applied to homœopathists. By F. H. Orme, M. D., Atlanta. Peprint from the New York *Medical Times.*

Aphasia with details of two interesting cases. By Philip Zenner, A. M., M. D., Cincinnati, O.

Address. By J. G. Thomas, M.D., Savannah, Ga. In defense of the National Board of Health against attacks in Congress, and on the importance of sapelo quarantine station as a place of refuge for dangerous and infected vessels for the South-Atlantic States.

Aphasia, With Details of Two Interesting Cases. By Philip Zenner, A.M., M.D., Cincinnati, O. Reprinted from the Cincinnati *Lancet and Clinic*, April 7, 1883.

Report of Proceedings of the Illinois State Board of Health. Quarterly Meeting, Chicago, April 12 and 14, 1883.

Proceedings of the Sanitary Council of the Mississippi Valley at its Fifth Annual Meeting, Jacksonville, Miss., April 3 and 4, 1883.

Another time we shall sieze an opportunity to comment freely on the texts supplied by both the above reports of proceedings. They present several points and facts of great interest to the profession at large, and to certain State Legislatures and Medical Colleges in particular.

